# Transfection of the IHH gene into rabbit BMSCs in a simulated microgravity environment promotes chondrogenic differentiation and inhibits cartilage aging

**DOI:** 10.18632/oncotarget.11871

**Published:** 2016-09-06

**Authors:** Peng-Cheng Liu, Kuan Liu, Jun-Feng Liu, Kuo Xia, Li-Yang Chen, Xing Wu

**Affiliations:** ^1^ Department of Orthopaedics, Shanghai Tenth People's Hospital, Tongji University, School of Medicine, Shanghai, People's Republic of China; ^2^ Department of Rehabilitation, Taihe Hospital, Hubei University of Medicine, Shiyan, Hubei, People's Republic of China

**Keywords:** BMSCs, hedgehog signaling pathway, Indian hedgehog, rotary cell culture system, cartilage tissue engineering, Gerotarget

## Abstract

The effect of overexpressing the Indian hedgehog (IHH) gene on the chondrogenic differentiation of rabbit bone marrow-derived mesenchymal stem cells (BMSCs) was investigated in a simulated microgravity environment. An adenovirus plasmid encoding the rabbit IHH gene was constructed *in vitro* and transfected into rabbit BMSCs. Two large groups were used: conventional cell culture and induction model group and simulated microgravity environment group. Each large group was further divided into blank control group, GFP transfection group, and IHH transfection group. During differentiation induction, the expression levels of cartilage-related and cartilage hypertrophy-related genes and proteins in each group were determined. In the conventional model, the IHH transfection group expressed high levels of cartilage-related factors (Coll2 and ANCN) at the early stage of differentiation induction and expressed high levels of cartilage hypertrophy-related factors (Coll10, annexin 5, and ALP) at the late stage. Under the simulated microgravity environment, the IHH transfection group expressed high levels of cartilage-related factors and low levels of cartilage hypertrophy-related factors at all stages of differentiation induction. Under the simulated microgravity environment, transfection of the IHH gene into BMSCs effectively promoted the generation of cartilage and inhibited cartilage aging and osteogenesis. Therefore, this technique is suitable for cartilage tissue engineering.

## INTRODUCTION

Cartilage tissue engineering provides new ideas for the treatment of cartilage damage and defects. Bone marrow-derived mesenchymal stem cells (BMSCs) are currently a recognized source of seed cells for cartilage tissue engineering [1–3]; however, there are still some deficiencies. Tissues constructed from BMSCs through cartilage tissue engineering still exhibit some histological, morphological, and biomechanical differences compared with normal cartilage tissues in the body [4]. In addition, cartilage cells or tissues constructed from BMSCs *in vitro* through cartilage tissue engineering are prone to aging and osteogenesis; therefore, they are not suitable for use in repairing cartilage damage or defects [5].

Indian hedgehog (IHH) is a conserved protein in the hedgehog signaling pathway and performs important regulatory functions in cartilage hypertrophy and bone formation. Defects in the IHH gene or protein result in the malformation of extremities, along with both a reduction in chondrocyte proliferation and swelling of hypertrophic chondrocytes [6]. IHH regulates many stages of chondrocyte proliferation and differentiation directly and indirectly. IHH is currently thought to promote increased parathyroid hormone/parathyroid-hormone-related peptide (PTH/PTHrP) expression in chondrocytes surrounding joints; the latter can interact with its receptor, parathyroid hormone receptor type 1 (Pthr-1), to promote the proliferation and inhibit the hypertrophy of chondrocytes. Furthermore, IHH and PTHrP form a negative feedback loop to decrease IHH secretion through the cAMP/PKA pathway [7]. Studies showed that transfection of IHH into BMSCs, either alone or in combination with bone morphogenetic protein-2 (BMP-2) or transforming growth factor-β (TGF-β), promoted chondrogenesis in primary BMSCs [8], which provided the theoretical basis for using IHH gene transfection to construct tissue-engineered cartilage.

In addition to nutritional support, the growth of chondrocytes also requires a specific growth environment, i.e., a microenvironment for cell growth. Conventional approaches for the *in vitro* culture and differentiation induction of BMSCs are usually limited to two-dimensional cell culture environments. Cells may present a phenomenon similar to “dedifferentiation”, in which they gradually lose many of the physiological characteristics of the original tissues [9]. In addition, cartilage tissues produced after induction can only approximate the histomorphology and chemical components of cartilage but cannot actually regenerate the original cartilage [4]. The rotary cell culture system (RCCS) is an unconventional three-dimensional microgravity culture system that allows culture materials to establish a suspension track, similar to a homogeneous fluid, on a horizontal axis. The gravity vector of the culture materials continues to distribute randomly; therefore, a continuous free-fall status can be maintained. In addition, the apparent effect of gravity on the culture materials is reduced to approximately 10^−2^ g; therefore, cells can overcome the effect of gravity and can easily aggregate and adhere to the surface of microcarriers. The RCCS can also provide specific biomechanical stimuli, such as shear force, joint compression force, and hydrostatic pressure, to build a microenvironment similar to that found inside the body, thus allowing cells to distribute more evenly on the scaffold material. This effect is helpful for the transport of nutrients and the removal of metabolic wastes, thus promoting chondrocyte proliferation and cartilage matrix synthesis [10, 11]. Studies have confirmed that RCCS promotes the differentiation of BMSCs into chondrocytes and is conducive to the maintenance of the chondrocyte phenotype and biomechanical characteristics [12].

The present study used an IHH adenovirus plasmid to transfect rabbit BMSCs to explore the effect of IHH gene transfection on the chondrogenic differentiation of BMSCs. Additionally, this system was combined with the RCCS system to investigate the feasibility of constructing tissue-engineered cartilage using IHH transfection of BMSCs under a simulated microgravity environment similar to the body to provide a theoretical basis for the repair of cartilage defects *in vivo*.

## RESULTS

### Morphology of BMSCs

**Figure 1:**
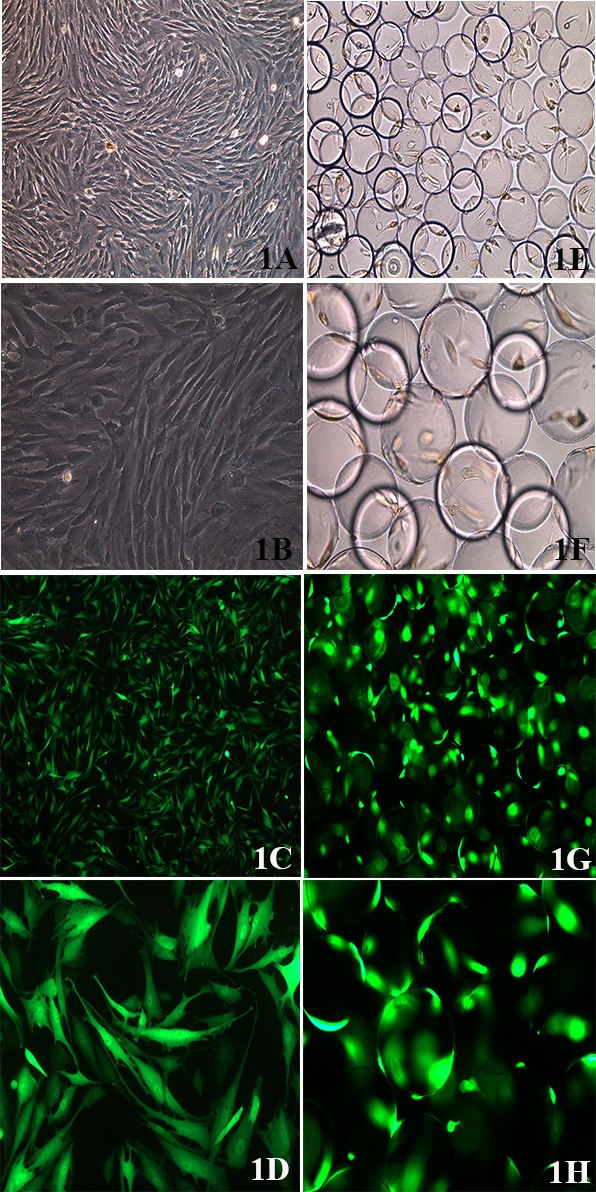
BMSC morphology **A.** BMSC morphology as observed on an inverted microscope (100X); **B.** (200X). **C.** BMSC morphology after viral transfection, as observed via fluorescence microscopy (100X), **D.** (200X). **E.** Adhesion of BMSCs on the surface of the Cytodex 3 microcarriers (100X), **F.** (200X). **G.** Adhesion of BMSCs on the surface of microcarriers after viral transfection. “Lantern-like” cell-microcarrier complexes were observed via fluorescence microscopy (100X), **H.** (200X).

BMSCs showed a fibroblast-like morphology when viewed on an inverted microscope. The cells showed swirl-like growth at low magnification. At high magnification, the cells mainly had long fusiform or spindle-like shapes; the cell nuclei had oval shapes and mostly mitotic activity (Figure 1A, 1B). The IHH gene adenovirus plasmid successfully transfected rabbit BMSCs, and a small amount of green fluorescence could already be observed 12 h after transfection. The green fluorescence was strongest at 48 h. The efficiency of viral transfection reached above 95%, as observed on an inverted fluorescence microscope. After transfection, BMSCs expressed high levels of GFP (Figure 1C, 1D), suggesting that the viral plasmid was constructed successfully and was able to transfect rabbit BMSCs with high efficiency.

In the RCCS group, BMSCs that adhered to the surface of Cytodex 3 microcarriers were placed in a RCCS container for cell culture and differentiation induction. Cytodex 3 is a type of spherical cell culture microcarrier, with a diameter of approximately 25-30 μm, whose surface is coated with one layer of collagen, which allows the adhesion of BMSCs. BMSCs attach and grow on the surface of Cytodex 3 and are suspended in culture medium, which increases the contact area between the cells and the culture medium and can facilitate cell metabolism and growth. The adhesion of BMSCs on spherical microcarriers was observed using an inverted microscope (Figure 1E, 1F). After viral transfection, the BMSCs expressed GFP, and “lantern-like” cell-microcarrier complexes could therefore be observed via fluorescence microscopy (Figure 1G, 1H).

### IHH protein expression in BMSCs after viral transfection

**Figure 2:**
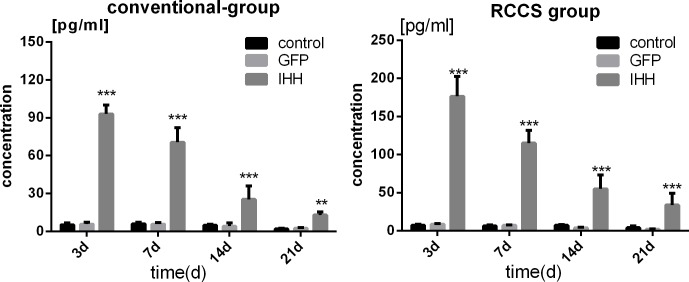
IHH protein expression during differentiation induction in BMSCs after viral transfection IHH protein expression levels in BMSCs after transfection with IHH viral plasmids in the conventional and RCCS groups and the comparison with the GFP transfection and blank control groups. “*” indicates a statistically significant difference (*P* < 0.05).

During the growth and differentiation of BMSCs, the activation of hedgehog signaling will promote increased IHH gene expression and expression of the IHH protein, which will be secreted by cells and interact with related receptors on the cell membrane, thus initiating a series of reactions [8]. The IHH protein concentration in culture medium reflects the activity of the IHH signaling pathway. The IHH protein expression was evaluated using ELISA; in both the conventional group and the RCCS group, the IHH protein was overexpressed in the IHH transfection group, and the expression on days 3, 7, 14, and 21 was significantly higher than the expression in the GFP transfection group and the control group (*P* < 0.05) (Figure 2). However, the IHH protein expression gradually decreased over time, consistent with the transient nature of adenoviral expression, thus indicating that the BMSCs were successfully transfected by the IHH gene adenovirus plasmid.

### Expression of cartilage- and cartilage hypertrophy-related genes during differentiation induction

Collagen II and aggrecan are characteristic proteins in the chondrocyte matrix. The SOX9 gene is closely associated with cartilage synthesis, and increased SOX9 transcription can significantly promote cartilage synthesis. Therefore, the collagen II, aggrecan, and SOX9 genes are considered cartilage synthesis-related genes, and their mRNA expression levels reflect the state of cartilage synthesis. Collagen X is a characteristic protein of the osteoblast matrix. ALP expression gradually increases with the gradual maturation, hypertrophy, and aging of chondrocytes during the process of endochondral ossification. Finally, as a marker of cell apoptosis, annexin V expression also increases during cartilage hypertrophy and aging [13]. In this experiment, the collagen X, ALP, and annexin V genes were used as chondrocyte hypertrophy- and aging-related genes, and their mRNA expression levels were determined using real-time PCR.

**Figure 3:**
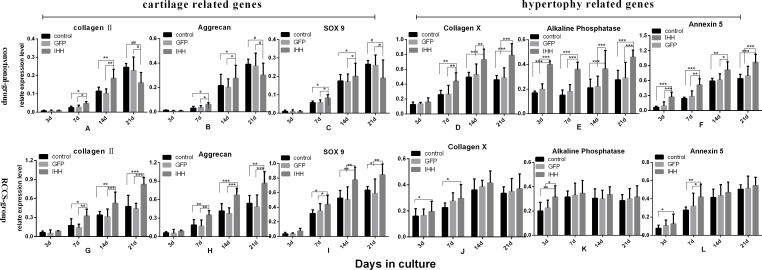
Expression of related genes during differentiation induction **A.**-**F.** Expression levels of related genes in the process of differentiation induction in the conventional group. **G.**-**L.** Expression levels of related genes in the RCCS group; (A-C) and (G-I) show the expression levels of cartilage-related genes; (D-F) and (J-L) show the expression levels of cartilage hypertrophy-related genes. “*” indicates a statistically significant difference (*P* < 0.05).

The results showed that in the conventional group, the expression of cartilage-related genes gradually increased in the control and GFP transfection groups during chondrogenic differentiation. In the IHH transfection group, the expression of related genes initially tended to increase and then decreased. The expression in the IHH group was slightly higher than that of the other two groups on days 7 and 14 of differentiation induction (*P* < 0.05) but was lower than that of the other two groups on day 21 (*P* < 0.05) (Figure 3A-3C). The expression levels of cartilage hypertrophy-related genes gradually increased in all groups, and the expression levels of related genes in the IHH transfection group were all higher than those in the control and GFP transfection groups (*P* < 0.05) (Figure 3D-3F).

In the RCCS group, the mRNA expression of cartilage-related genes such as collagen II, aggrecan, and SOX9 gradually increased during differentiation induction; the expression levels of related genes at different stages of differentiation induction in the IHH transfection group were all significantly higher than those in the control and GFP transfection group (*P* < 0.05) (Figure 3G-3I). The mRNA expression levels of cartilage hypertrophy-related genes such as collagen X, ALP, and annexin V gradually increased and then became stable at the middle stage of differentiation induction; the expression levels even decreased at later stages. The expression levels of cartilage hypertrophy-related genes in the IHH transfection group at the early stage of differentiation induction were slightly higher than those in the other two groups; however, at the late stage of differentiation induction, there was no difference from the other two groups (Figure 3J-3L).

### Expression of cartilage-related proteins during differentiation induction

**Figure 4:**
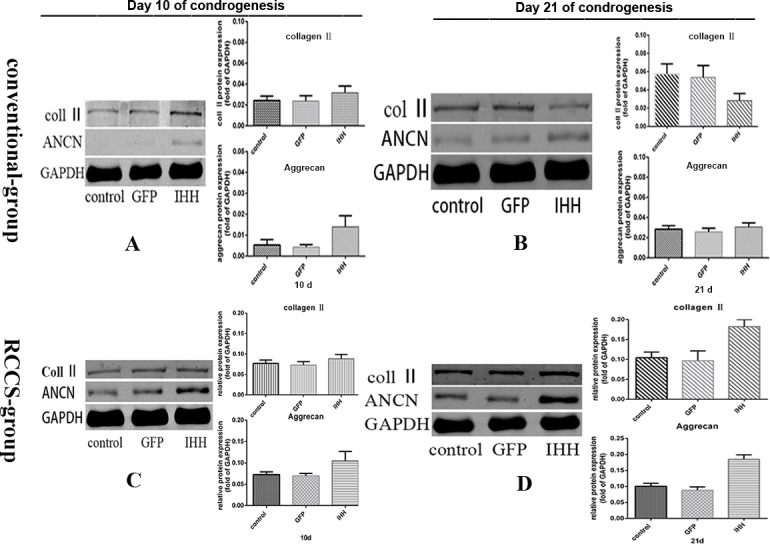
Expression of collagen II and aggrecan during differentiation induction **A.**, **B.** Expression of cartilage-related proteins in the conventional group; **C.**, **D.** Expression of cartilage-related proteins in the RCCS group.

The expression of cartilage-related proteins during differentiation induction was evaluated via Western blotting. The results showed that on day 10 after differentiation induction in the conventional group, the protein expression levels of cartilage-related collagen II and aggrecan were both low. However, the protein expression levels in the IHH transfection group were slightly higher than those in the control group and the GFP transfection group (*P* < 0.05) (Figure 4A). On day 21 after differentiation induction, the expression level of collagen II in the IHH transfection group was significantly lower than in the GFP transfection group and the control group (*P* < 0.05) (Figure 4B); however, aggrecan expression did not show a significant difference among all the groups (*P* > 0.05) (Figure 4B).

In the RCCS condition, the expression levels of collagen II and aggrecan were higher than in the conventionally cultured cells. On days 10 and 21 after differentiation induction, the protein expression levels in the IHH transfection group were significantly higher than those in the other groups (*P* > 0.05) (Figure 4C, 4D).

### Toluidine blue and annexin V-Cy3 staining

After chondrogenic differentiation, the cell morphology of BMSCs gradually changed from fusiform or spindle shapes to oval or round and gradually exhibited a chondrocyte-like morphology [14]. After toluidine blue staining, the extracellular matrix stains a light blue color, and cell nuclei show dark staining. As more extracellular matrix is synthesized, the staining results tended to be dark blue [8]. When chondrocytes become hypertrophic or apoptotic, annexin V is expressed and appears as red fluorescence via fluorescence microscopy after annexin V-Cy3 immunofluorescence staining. Stronger red staining reflects higher levels of annexin V expression [13].

**Figure 5:**
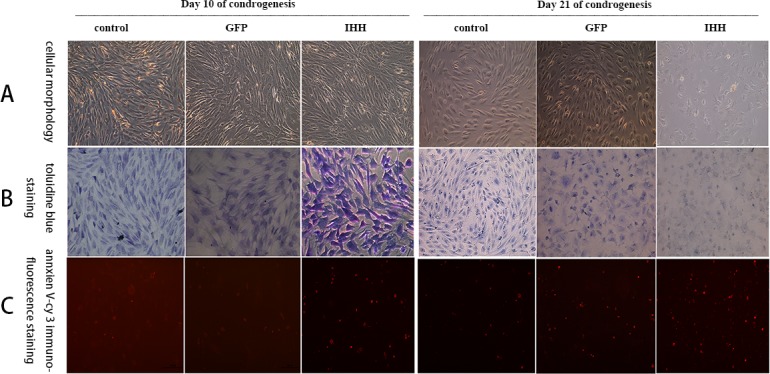
Staining results for the process of differentiation induction in the conventional group **A.** Morphological changes of cells during differentiation induction; **B.** Toluidine blue staining; **C.** Annexin V-Cy3 immunofluorescence staining.

The results showed that the morphology of BMSCs started to change on day 10 after differentiation induction in the conventional group; cells gradually adopted a short fusiform or oval shape compared with their original long fusiform shape. The changes in cell morphology in the IHH transfection group were the most obvious (Figure 5A, 10 days). The toluidine blue staining results in all groups were lighter and the staining in the IHH group was darker than that in the other groups (Figure 5B, 10 days). The annexin V-Cy3 staining results showed that all groups displayed a small amount of red fluorescence. The red fluorescence in the IHH group was stronger, indicating that annexin V expression was higher than in the other two groups (Figure 5C, 10 days). On day 21 after differentiation induction, the morphology of the BMSCs gradually exhibited an oval or round shape. However, cells in the IHH transfection group had significantly increased refraction on the cell border, a small number of vacuoles were observed in the cytoplasm, and there was a small number of apoptotic cells (Figure 5A, 21 days). The toluidine blue staining results in all groups showed a light blue color; however, the staining was weaker in the IHH transfection group (Figure 5B, 21 days). The annexin V-Cy3 staining results showed that all groups had stronger red fluorescence, while the expression of red fluorescence in the IHH transfection group was stronger than that in the other groups (Figure 5C, 21 days).

**Figure 6:**
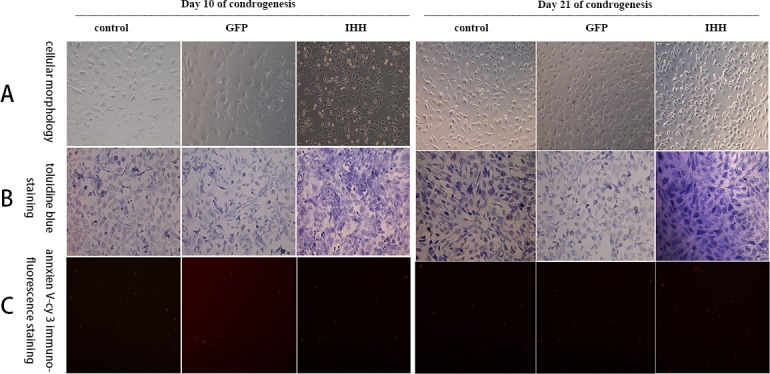
Staining results for the process of differentiation induction in the RCCS group **A.** Morphological changes of cells during differentiation induction; **B.** Toluidine blue staining; **C.** Annexin V-Cy3 immunofluorescence staining.

In the RCCS differentiation induction condition, toluidine blue staining and annexin V-Cy3 immunofluorescence staining showed that on day 10 after differentiation induction, BMSC morphology showed significant changes, and cells displayed an oval or round morphology similar to that of chondrocytes (Figure 6A, 10 days). All groups showed light blue color after toluidine blue staining, and the staining in the IHH transfection group was stronger, with a darker color, than that in the other groups (Figure 6B, 10 days). The annexin V-Cy3 staining results showed that on day 10 after differentiation induction, the expression of red fluorescence in all the groups was weaker, and there was no significant difference among all the groups (Figure 6C, 10 days). On day 21 after differentiation induction, cell morphology in all the groups was significantly altered and displayed a cobblestone-like oval or round cell morphology (Figure 6A, 21 days). Toluidine blue staining showed blue color in all groups; the color in the IHH transfection group was significantly darker than in the control group and the GFP group, indicating the rich synthesis of cartilage matrix (Figure 6B, 21 days). The annexin V-Cy3 staining results showed that the expression of fluorescence was lower in all groups, and there was no significant difference among the groups (Figure 6C, 21 days).

### ALP activities in the supernatant of cell culture medium

**Figure 7:**
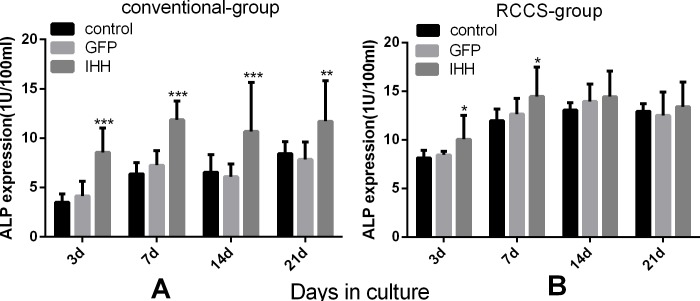
Evaluation of ALP activity during differentiation induction **A.** ALP activity levels in the culture supernatant from each group of the conventional group; **B.** ALP activity levels in the culture supernatant from each group of the RCCS group. “*” indicates a statistically significant difference (*P* < 0.05).

ALP expression during differentiation induction also reflects the condition of cartilage hypertrophy and aging or osteogenesis. The ALP activity in culture medium or the cells was determined using ELISA. The results showed that the ALP activity in culture medium gradually increased during differentiation in all groups of the conventional group. In addition, ALP activity in the IHH transfection group was higher at all stages of differentiation induction compared with the control group and the GFP group (Figure 7A). In the RCCS group, ALP activity measurement showed that at the initial stage of differentiation induction, ALP activity in the IHH group was slightly higher than that in the other two groups (P < 0.05); however, at the late stage of induction, the differences among all the groups were not significant (*P* > 0.05) (Figure 7B).

## DISCUSSION

Chondrogenesis in BMSCs is regulated by a variety of signaling pathways acting together. For example, the TGF-β/BMP, Wnt, FGF, IGF, and hedgehog pathways all play different regulatory roles in this process. The Hedgehog signaling pathway is one of the most important signaling pathways [15, 16]. IHH is a homolog of the Hedgehog signaling pathway. It regulates cartilage development through direct and indirect pathways; it can simultaneously promote chondrocyte proliferation and inhibit chondrocyte aging. The feedback loop formed between IHH and its receptor, PTHrP, plays an important role in IHH signaling [7]. Some basic studies have also shown that when transfection into BMSCs either alone or with TGF-β or BMP-2, IHH promoted the differentiation of BMSCs into chondrocytes [8]. Under conventional cell culture and induction conditions, the expression levels of cartilage-related genes in the IHH transfection group increased initially and then decreased; in addition, expression levels on day 21 were significantly lower than those in the other groups. However, the expression levels of chondrocyte hypertrophy-related genes gradually increased and were higher than those in the control group (*P* < 0.05) (Figure 3A-3F). This phenomenon was also confirmed by the subsequent protein expression results. Although the collagen II and aggrecan protein expression levels on day 10 were higher than those in the other two groups, the expression levels on day 21 were lower than those in the non-transfected group and the GFP transfection group. Annexin V not only reflects the level of cell apoptosis but is also a maker for chondrocyte hypertrophy and aging [13]. Annexin V-Cy3 staining in cells grown on slides showed that the IHH transfection group exhibited stronger red fluorescence, indicating that the IHH transfection group had severe chondrocyte hypertrophy or aging after induction.

The above results indicated that in the conventional two-dimensional cell culture and induction model, although the IHH gene facilitated the progression of cartilage differentiation at early stages of differentiation induction, it also promoted cartilage hypertrophy, aging, and even apoptosis. When the IHH signal is highly expressed, the progression of endochondral ossification is accelerated, and the rapidly proliferated chondrocytes will undergo differentiation, aging, or even apoptosis [8]. In this experiment, the expression levels of cartilage-related genes and cartilage marker proteins at the early stage of differentiation induction in the IHH transfection group was better than that in the control group; however, the levels were significantly lower than those in the control group at the late stage, which also explained this question. Furthermore, the IHH transfection group expressed high levels of annexin V, which also confirmed that these chondrocytes underwent aging. Fischer J et al [17] showed that intermittent PTHrP protein stimulation can promote chondrogenesis in mesenchymal stem cells (MSCs) and that the expression levels of cartilage-related collagen II and proteoglycan significantly increased; however, constant PTHrP protein stimulation suppressed chondrogenesis, and the expression of cartilage hypertrophy-related marker genes and proteins did not increase. These results also suggest that the accumulation of excessive IHH/PTHrP signals after IHH gene transfection might accelerate chondrogenesis, chondrocyte maturation, hypertrophy, and endochondral ossification in BMSCs.

In addition, during differentiation induction in BMSCs, the expression levels of the collagen X and ALP genes increased, and the expression levels of these genes were significantly higher in the IHH transfection group than in the control and non-transfection groups. These results indicated that the increased IHH signaling stimulated the activation of cartilage hypertrophy- or osteogenesis-related genes and signaling pathways. The latter may cause collagen II outside the chondrocytes to gradually become osteoblast-related collagen X and promote ALP secretion. The results showed that during differentiation induction, ALP expression in the IHH transfection group increased and was higher than that in the control group. These results also reflected the activation of related signaling pathways. The presence of the cAMP/PKA pathway activator forskolin was effective in reproducing the effect of constant PTHrP stimulation on the inhibition of chondrogenesis and the promotion of chondrocyte hypertrophy and maturation [17]. These results indicated that the cAMP/PKA pathway plays an important role as an intermediate signaling regulator in this process.

In RCCS, cells attach to the three-dimensional scaffold material and can freely rotate with the rotation of the base. Therefore, the culture materials can establish a suspension track similar to a homogeneous fluid on a horizontal axis. The gravity vector continues to distribute randomly; therefore, the culture materials can be continuously maintained in free-fall, which facilitates cell aggregation and thus enhances cell-cell and cell-matrix communication. The latter can increase SOX9 transcription through the activation of SIRT1, thus increasing the expression of the downstream target gene collagen II [10, 18]. The RCCS cell culture container has a silicone gas exchange membrane to permit adequate gas exchange by cells or tissues. This highly efficient material transfer inside and outside cells and among cells enables cells to have a stronger proliferation and differentiation capacity [11, 19]. In addition, RCCS can provide some types of biomechanical stimuli, such as shear force, joint compression force, and hydrostatic pressure, to mimic the microenvironment inside the body to allow cells to distribute more evenly on the scaffold material. This property is helpful for the transport of nutrients and the removal of metabolic waste, thus promoting chondrocyte proliferation and cartilage matrix synthesis. We inoculated rabbit BMSCs on microcarriers, which were then cultured in RCCS and were induced to undergo chondrogenesis. The results showed that the effect of IHH gene transfection on the chondrogenesis of BMSCs under RCCS induction conditions was different from that observed under the conventional model. Specifically, the expression of cartilage-related genes, such as collagen II, aggrecan, and SOX9, as well as the related proteins, gradually increased during differentiation induction, and the expression levels in the IHH transfection group were significantly higher than those in the control and GFP transfection groups. However, the expression levels of cartilage hypertrophy-related markers were lower, and there was no significant difference among the groups. These results indicated that under the RCCS induction condition, IHH overexpression promoted the chondrogenesis of BMSCs and effectively inhibited chondrocyte hypertrophy.

RCCS can provide some types of biomechanical stimuli for the cultured materials. These stimuli can mediate changes in related signaling pathways, thus further influencing the chondrogenesis of BMSCs. Under some biomechanical conditions, the SOX9, Runx2, and p38 MAPK expression levels increased in pluripotent stem cells, and this change was closely associated with the synthesis of cartilage [20]. The integrin, cytoskeletal, and MAPK signaling pathways are some of the most important pathways for cells to receive and transduce mechanical signals. Integrins can sense extracellular mechanical signals and transform them into chemical signals, which are then integrated into intracellular signals; thus, a series of cell biological behaviors are regulated through signaling pathways, such as the cytoskeletal signaling and MAPK pathways [21]. Studies have shown that the expression of integrin β1 in MSCs increased as a function of microgravity; however, the expression of the downstream signals FAK and PYK2 significantly decreased. In addition, the phosphorylation of Ras and ERK was also inhibited, phosphorylated ERK directly regulated Runx2 transcription, and Runx2 was closely associated with osteogenesis of MSCs [22, 23]. Therefore, under the effect of microgravity, the reduction of FAK and PYK2 phosphorylation and the disorder of the MAPK/ERK signaling pathway might promote MSCs to become osteoblasts after the inhibition of MSC chondrogenesis. In this experiment, the expression levels of osteogenesis-related genes, collagen X and ALP, were lower and the ALP activity in the culture medium was also lower, which might be associated with the inhibition of osteogenesis after the BMSCs differentiated into chondrocytes. However, the specific signaling mechanism involved still requires further exploration.

An adenoviral expression plasmid containing the rabbit IHH gene was successfully constructed *in vitro*. This plasmid was transfected into BMSCs at high efficiency to overexpress the rabbit IHH protein. Under conventional cell culture and induction conditions, BMSCs transfected with the IHH gene exhibited cartilage synthesis at the early stage of differentiation induction; however, cartilage hypertrophy, aging, and osteogenic differentiation were also present immediately. Conversely, under RCCS culture and induction conditions, IHH gene transfection into BMSCs effectively promoted cartilage synthesis and inhibited cartilage aging and osteogenic differentiation, demonstrating the suitability of this method for the needs of cartilage tissue engineering.

## MATERIALS AND METHODS

### BMSC culture and grouping

Second-generation Rabbit BMSCs (Mesenchymal Stem Cells, Lot Number: 140705J31, Certificate of Analysis can be download from website of Cyagen Biosciences Inc. http://www.cyagen.com/media/uploads/QAPI0814A0_RBXMX-01001_CoA_140705J31.pdf) were purchased from Cyagen Biosciences Inc. (Catalog No. RBXMX-01001, Cyagen Biosciences Inc., China) and were continuously subcultured in BMSC-dedicated cell culture medium (L-DMEM containing 10% FBS, 1% PS, and 1 ng/ml recombinant human fibroblast growth factor-2 (rhFGF)). BMSCs at passage 2 were used for viral transfection and induction of chondrogenesis. The culture medium for chondrogenesis consisted of H-DMEM containing 10^−7^ M dexamethasone, 50 μg/ml ascorbate, sodium pyruvate, L-proline, 10% ITS solution, and 10 ng/ml transforming growth factor-β (TGF-β). The experiment was divided into two large groups: the RCCS group and the conventional group. Each large group was further divided into three small groups: the IHH gene adenovirus plasmid transfection group, the GFP adenovirus plasmid transfection group, and the blank control group. In the RCCS group, the chondrogenesis of all cells was performed in the simulated microgravity environment. In the conventional group, the cell culture and chondrogenesis of all cells were performed in 6-well plates.

### Construction of recombinant IHH adenovirus plasmids

The pDC316-mCMV-EGFP (Qiagen 12163, Genopure Plasmid Maxi Kit, Qiagen, Germany) was used as a shuttle plasmid for the backbone vector and enzymatic digestion. Primers were designed and synthesized to amplify the fragment of the target gene, rabbit IHH (Gene ID: 100008942). The fragment was ligated into the XbaI/XhoI-digested overexpression vector. The ligated product was transformed into competent cells, and single colonies were identified by sequencing. The clone with the correct sequence was the overexpression plasmid containing the target gene. The constructed adenovirus overexpression plasmid and the backbone plasmid were co-transfected into HEK293 cells for viral packaging. The virus stock solution was collected, and the titer of the virus solution was determined using UV spectrophotometry. The viral solution was concentrated to 10^11-12^ pfu/ml by ultrafiltration concentration. The adenovirus overexpression plasmid containing the GFP gene was purchased from Shanghai GenomeDitech (China), and the titer was 1*10^11^ pfu/ml.

### Transfection of adenovirus plasmids into rabbit BMSCs and induction of chondrogenic differentiation

Upon reaching 70%-80% confluence, rabbit BMSCs were transfected with either the IHH gene adenovirus plasmid or the GFP adenovirus plasmid at 200 pfu/cell of active virus particles. After 2 h of infection, the medium was replaced with fresh BMSC cell culture medium. After 24 h of infection, cells from each group were collected using 0.25% trypsin-EDTA solution and then counted. For the RCCS group, transfected cells at 4*10^5^ cells/ml, the cell culture microcarrier Cytodex 3 at 5 mg/ml, and BMSC complete culture medium were mixed thoroughly and inoculated into an RCCS culture container. The container was then connected to a rotating base and placed in a CO_2_ incubator for culture. For the conventional group, transfected cells were inoculated onto 6-well plates at a density of 3*10^5^ cells/well. A total of 2 ml of BMSC complete culture medium was added to each well, and cells were evenly dispersed by repeatedly moving the plates back and forth and left to right. Cells were cultured in a CO_2_ incubator. For all groups, the culture medium was replaced with chondrocyte differentiation medium after 24 h, after which the culture medium was replaced once every 2-3 days for 21 days to perform chondrogenic differentiation.

Cell inoculation and sampling in the RCCS group were performed as described by Wu X et al [12]. Cells were harvested, and cell density was adjusted to 10^5^ cells/ml. Sterilized Cytodex 3 microcarriers were washed with BMSC culture medium and suspended in an appropriate amount of complete culture medium to adjust the concentration to 5 mg/ml. The cell suspension and Cytodex 3 were fully mixed to achieve a final cell density and microcarrier concentration of 4*10^5^ cells/ml and 5 mg/ml, respectively. The mixture was then inoculated into 50-ml or 10-ml RCCS cell culture containers. After being installed on the rotating base, the containers were placed in a 5% CO_2_ incubator for culture. The rotation speed of the RCCS container was adjusted to approximately 10-12 rpm to allow full contact between the cells and the microcarriers. After 24 h, the rotation speed of the container was adjusted to approximately 12-14 rpm such that the culture material in the container no longer came in contact with the container wall and remained in free-fall during rotation. When samples needed to be removed from the container for evaluation, the power was first turned off, and the container was then placed in a biosafety cabinet for manipulation. The container was tilted to allow the precipitation of the culture materials to the bottom of the container. The supernatant was collected to determine the IHH protein concentration and alkaline phosphatase (ALP) activity in the culture medium. When cells needed to be harvested, the culture materials in the container were mixed thoroughly, and an appropriate amount of cells-microcarriers-culture medium was aspirated using a syringe. Samples were washed 2-3 times with PBS, and the cells were dissociated from the microcarriers using 0.25% trypsin-EDTA. Cells were harvested for protein and RNA extraction or for inoculation onto slides for toluidine blue staining or annexin V immunofluorescence staining.

### Measurement of IHH protein expression and ALP activity

After BMSCs were transfected with the IHH viral plasmids for 24-48 h, the GFP expression in each group of cells was observed via fluorescence microscopy. On days 3, 7, 14, and 21 after differentiation induction, 24-h cell culture supernatant was collected. Three samples from each group were randomly selected to determine the IHH protein concentrations in the cell culture medium using ELISA (rabbit IHH protein ELISA kit, Laibio, China) according to the method of Steinert AF et al [8]. In addition, the ALP activity in the cell culture medium was determined using ELISA according to the instructions of the ALP activity assay kit. Three samples were randomly selected from each group. The ALP protein standard or 5 μl of the samples, 50 μl of buffer solution, 50 μl of substrate, and 5 μl of double-distilled water were fully mixed in a 96-well plate and incubated for 15 min in a 37°C water bath. The absorbance values of the samples in each group were determined using spectrophotometry at 520 nm. The ALP activity of each sample was calculated according to the formula in the instructions of the ALP activity assay kit.

### RNA extraction and reverse transcription analysis

On days 3, 7, 14, and 21 after differentiation induction, three samples were randomly selected from each group for RNA extraction and RT-PCR analysis. Cell samples were harvested from each group and mixed with 1 ml of Trizol reagent (Invitrogen, USA). Reagents, such as chloroform, isopropanol, and anhydrous ethanol, were added separately to extract and purify RNA from cells. The concentration and purity of the extracted RNA were determined spectrophotometrically based on the absorbance at 260 nm. RT-PCR was performed using 50 μg of RNA from each sample to synthesize cDNA. The 10-μl reaction contained 1 μl of Oligo dT Primer (50 μM), 1 μl of dNTP Mixture (10 mM each), 50 μg of total RNA, and RNase-free double-distilled water. The method was performed according to the instructions of the PrimeScript^TM^ RT-PCR kit (TaKaRa, China).

The concentration of synthesized cDNA was determined using spectrophotometry. The expression of related genes in each sample was analyzed according to the instruction manual of the Real-time PCR kit (KaPa, USA). The 20-μl reaction contained 10 μl of 2X qPCR master mix, 0.4 μl of 10 μM forward primer, 0.4 μl of 10 μM reverse primer, 2 μl of cDNA, 0.4 μl of 50X ROX high/low, and enough ddH_2_O for a final volume of 20 μl. The evaluated cartilage-related genes included collagen II, aggrecan (ANCN), and sex determining region Y-box 9 (SOX 9). The evaluated cartilage hypertrophy-related genes mainly included collagen X, alkaline phosphatase (ALP), and annexin V. In addition, GAPDH was selected as the internal control. Two primers were synthesized for each gene based on the gene sequence information at NCBI. The sizes and nucleotide sequences of the related primers are shown in Table 1.

**Table 1:** The sizes and nucleotide sequences of the related primers

Gene name	Product fragment size	Sequence (5′ to 3′)
Chondrogenic markers		
Col II	276 bp	Forward: GCTCCCAGAACATCACCTACCA Reverse: ATTCCTGCTCAGGCCCTCC
ANCN	127 bp	Forward: ATGGCTTCCACCAGTGCG Reverse: CGGATGCCGTAGGTTCTCA
SOX9	250 bp	Forward: CTCGAAACCGACTGGCAACT Reverse: AACAAGCGGTCCAAAGGAAA
Hypertrophy and osteogenic markers		
Col X	103 bp	Forward: CCCTTCTGCTGCTAGTGTC Reverse: GTCTTGGTGTTGGGTTGTG
ALP	155 bp	Forward: CCTCTTGGGTCTCTTTGAGC Reverse: CAATCCTGCCTCCTTCCA
Annexin V	110 bp	Forward: GCAGAACTAACAGCCATAA Reverse: AGAACCACCAACATCCTC
Internal control gene		
GAPDH	140 bp	Forward: CCACTTTGTGAAGCTCATTTCCT Reverse: TCGTCCTCCTCTGGTGCTCT

### Protein extraction and evaluation of cartilage-related protein expression

On days 10 and 21 after differentiation induction, three samples were randomly selected from each group to extract cellular proteins. GAPDH was used as the internal control protein. The expression of cellular Coll II and ANCN was determined using Western blotting according to Wu X et al [24]. For the evaluation of Coll II expression, the primary antibody was a rabbit polyclonal antibody (Abcam, USA), and the secondary antibody was a goat anti-rabbit monoclonal antibody (Thermo, USA). The primary antibody for ANCN was a mouse monoclonal antibody (Thermo, USA), and the secondary antibody was a goat anti-mouse monoclonal antibody (Thermo, USA).

### Toluidine blue staining and annexin V-Cy3 immunofluorescence staining

On days 10 and 21 after differentiation induction, cells grown on slides were harvested for toluidine blue staining and annexin V staining. Toluidine blue can be used for non-specific staining to indicate cartilage synthesis, and the staining was performed according to the instruction manual of the chondrocyte toluidine blue staining reagent kit (Sigma, USA).

Annexin V not only is one of the characteristic makers of cell apoptosis but also reflects the status of chondrocyte hypertrophy and aging [13]. In this experiment, annexin V was used as a cartilage hypertrophy- and aging-related factor. During the differentiation induction in all groups, annexin V-Cy3 immunofluorescence staining was performed to analyze the chondrocyte hypertrophy and aging during the differentiation induction process. Staining was performed according to the instruction manual of the annexin V-Cy3 immunofluorescence staining reagent kit (Enzo Life Sciences, USA) and Steinert A F et al [8].

### Statistical methods

Measurement data are presented as $\bar{x}$ ± s. Statistical analysis was performed using SPSS 20.0 software. Comparison of mean values between two samples was performed using the two independent samples t-test. *P* < 0.05 indicated a statistically significance difference.
